# MACC1 mediates acetylcholine-induced invasion and migration by human gastric cancer cells

**DOI:** 10.18632/oncotarget.7634

**Published:** 2016-02-23

**Authors:** Ting Yang, Wanming He, Fei Cui, Jianling Xia, Rui Zhou, Zhenzhen Wu, Yang Zhao, Min Shi

**Affiliations:** ^1^ Department of Oncology, Nanfang Hospital, Southern Medical University, Guangzhou, China

**Keywords:** acetylcholine, MACC1, gastric cancer, invasion, migration

## Abstract

The neurotransmitter acetylcholine (ACh) promotes the growth and metastasis of several cancers via its M3 muscarinic receptor (M3R). Metastasis-associated in colon cancer-1 (MACC1) is an oncogene that is overexpressed in gastric cancer (GC) and plays an important role in GC progression, though it is unclear how MACC1 activity is regulated in GC. In this study, we demonstrated that ACh acts via M3Rs to promote GC cell invasion and migration as well as expression of several markers of epithelial-mesenchymal transition (EMT). The M3R antagonist darifenacin inhibited GC cell activity in both the presence and absence of exogenous ACh, suggesting GC cells secrete endogenous ACh, which then acts in an autocrine fashion to promote GC cell migration/invasion. ACh up-regulated MACC1 in GC cells, and MACC1 knockdown using siRNA attenuated the effects of ACh on GC cells. AMP-activated protein kinase (AMPK) served as an intermediate signal between ACh and MACC1. These findings suggest that ACh acts *via* a M3R/AMPK/MACC1 signaling pathway to promote GC cell invasion/migration, which provides insight into the mechanisms underlying GC growth and metastasis and may shed light on new targets for GC treatment.

## INTRODUCTION

Emerging evidence indicates that nerves play significant roles in the growth and spread of cancers [1-3]. For example, Zhao et al. showed that denervation of the stomach through vagotomy or local injection of neurotoxic agents suppresses gastric tumor development and progression [2]. The neurotransmitter of the vagus nerve, acetylcholine (ACh), is one of the classical neurotransmitters of the central and peripheral nervous systems and reportedly participates in the progression of some types of cancer [4, 5].

ACh acts via nicotinic and muscarinic receptors (nAChR and mAChR, respectively). Among the five mAChR subtypes (M1-M5), the M3 receptor (M3R) appears to play a pivotal role in cancer cell proliferation [6-8] and invasion [9, 10]. However, the metastatic effects of ACh in human gastric cancer (GC) remain far from clear. This prompted us to investigate whether ACh acting via M3Rs can stimulate GC cell invasion, migration and/or epithelial-mesenchymal transition (EMT) and the mechanism involved.

Metastasis-associated in colon cancer-1 (MACC1) is an oncogene first discovered in colon cancer [11], where its main action was thought to be promotion of metastasis. MACC1 was subsequently shown to be overexpressed in many other cancers [12-15], including GC [16]. Previous studies in our laboratory verified that patients exhibiting high MACC1 expression have poor outcomes, and that MACC1 enhances GC cell proliferation, invasion and migration *in vitro* and *in vivo* [16]. AMP-activated protein kinase (AMPK) plays a central role in the regulation of cellular metabolism and the maintenance of energy homeostasis in mammalian tissues [17, 18]. We demonstrated that MACC1 expression is significantly up-regulated following AMPK phosphorylation (activation) in response to glucose deprivation-induced metabolic stress [19]. The regulators upstream of AMPK phosphorylation remain unknown, however. Our aim in the present study was to determine whether ACh promotes GC cell invasion/migration and EMT via a M3R/AMPK/MACC1 signaling pathway.

## RESULTS

### ACh promotes GC cell invasion and migration and induces EMT progression

**Figure 1:**
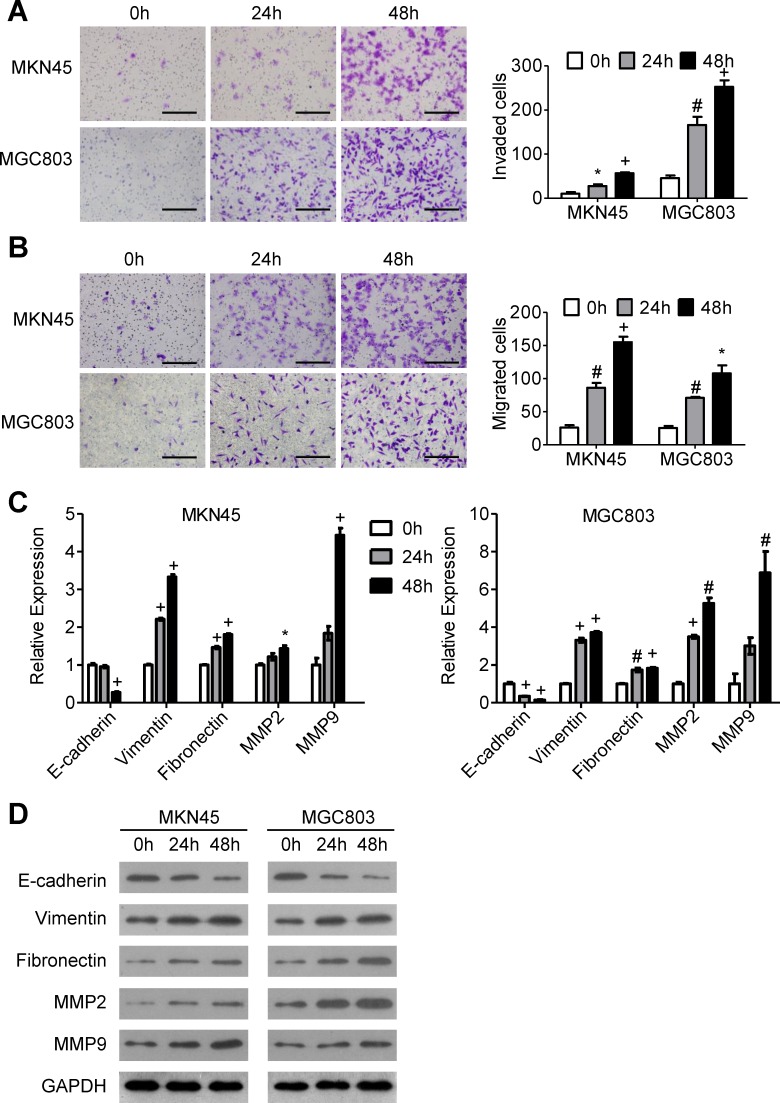
The effects of ACh on GC cell invasion, migration and EMT MKN45 and MGC803 cells were incubated with ACh (10 μM) for the indicated times. (A, B) Invasion **A.** and migration **B.** of MKN45 and MGC803 cells in transwell assays (scale bar = 200 μm). **C.** qRT-PCR analysis showing relative mRNA expression of the EMT markers E-cadherin, vimentin, fibronectin, MMP2 and MMP9. mRNA levels were normalized to the level of GAPDH mRNA. **D.** Western blots showing the protein expression of the indicated EMT markers. Quantitative data are presented as the mean±SEM from three independent experiments. *P < 0.05, ^#^*P* < 0.01, ^+^*P* < 0.001.

We stimulated MKN45 and MGC803 GC cells with 10 uM ACh for 0 h, 24 h or 48 h and then carried out invasion/migration assays. The results show that the number of invading and migrating cells increased in a time-dependent manner (Figure 1A and 1B). During the same period, ACh increased the mRNA and protein expression of vimentin, fibronectin, MMP2 and MMP9 and decreased expression of E-cadherin (Figure 1C and 1D), suggesting that ACh promotes EMT progression. On the other hand, ACh induced no significant morphological changes in GC cells (Supplementary Figure 1). These results indicate that ACh promotes the invasion/migration of GC cells and contributes to EMT progression.

### M3Rs mediate the effect of ACh on GC cell invasion/migration and EMT

**Figure 2:**
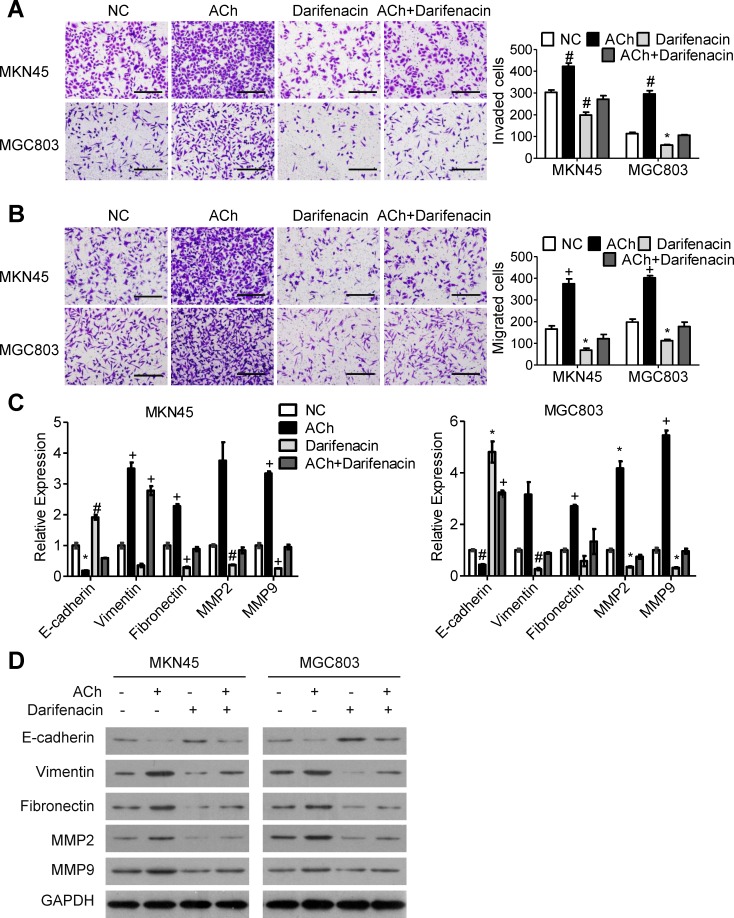
M3Rs mediate the effects of ACh on GC cell invasion, migration and EMT MKN45 and MGC803 cells were pretreated with darifenacin (10 μM) before incubation with ACh (10 μM), PBS was used as a negative control (NC). (A, B) Invasion **A.** and migration **B.** of MKN45 and MGC803 cells in transwell assays (scale bar=200 μm). **C.** qRT-PCR analysis showing relative mRNA expression of the indicated EMT markers. mRNA levels were normalized to the level of GAPDH mRNA. **D.** Western blots showing the protein expression of the indicated EMT markers. Quantitative data are presented as the mean±SEM from three independent experiments. Experimental groups were compared to the negative control group. *P < 0.05, ^#^*P* < 0.01, ^+^*P* < 0.001.

To investigate the role of M3Rs in ACh-induced invasion/migration, we pretreated GC cells with 10 μM darifenacin, a selective M3R antagonist, and then stimulated the cells with ACh or left them untreated. M3R blockade markedly reduced ACh-induced invasion/migration (Figure 2A and 2B) while decreasing ACh-induced expression of E-cadherin and increasing expression of vimentin, fibronectin, MMP2 and MMP9 (Figure 2C and 2D). Notably, when compared to a negative control, darifenacin also inhibited GC cell invasion/migration and EMT in the absence of exogenous ACh.

### MACC1 is essential for ACh-induced GC cell invasion/migration and EMT

**Figure 3:**
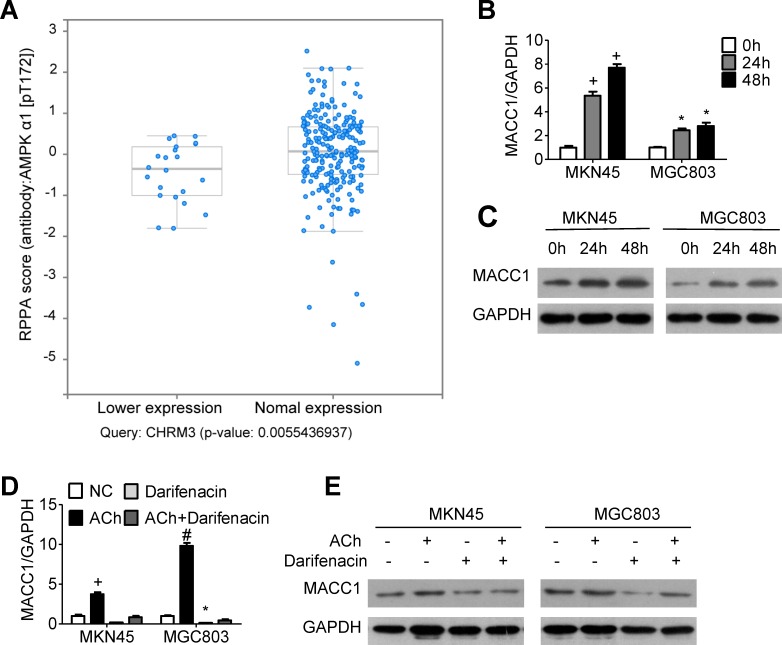
MACC1 expression is regulated by ACh and M3R **A.** Stomach adenocarcinoma data from the TCGA database was retrieved and analyzed using a tool in http://www.cbioportal.org/. Each blue dot represents one case of stomach adenocarcinoma. AMPK phosphorylation at T172 correlated positively with expression of CHRM3 mRNA (encoding M3R). (B, C) Effects of ACh on expression of MACC1 mRNA **B.** mRNA and protein **C.** detected using qRT-PCR and western blotting, respectively. MKN45 and MGC803 cells were pretreated with the M3R inhibitor darifenacin (10 μM) before ACh. (D, E) Effect of darifenacin on ACh-induced expression of MACC1 mRNA **D.** and protein **E.**. Quantitative data were presented as the mean±SEM from three independent experiments. Experimental groups were compared to the negative control group. *P < 0.05, ^#^*P* < 0.01, ^+^*P* < 0.001.

**Figure 4:**
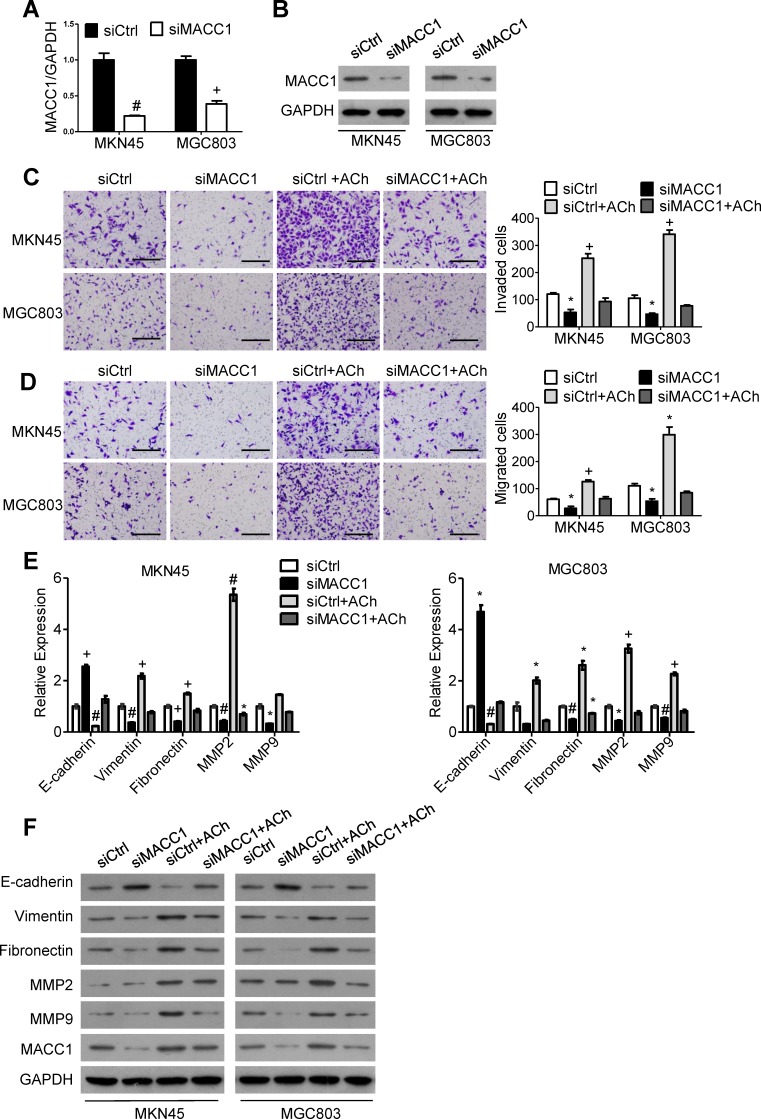
MACC1 mediates ACh promotion of GC cell invasion, migration and EMT (A, B) Efficiency of MACC1 knockdown was determined using qRT-PCR **A.** and western blotting **B.**. (C, D) Invasion **C.** and migration **D.** by MKN45 and MGC803 cells in transwell assays (scale bar=200 μm). **E.** qRT-PCR analysis showing relative mRNA expression of the indicated EMT markers. mRNA levels were normalized to the level of GAPDH mRNA. **F.** Western blots showing the protein expression of the indicated EMT markers. Quantitative data were presented as the mean±SEM from three independent experiments. Experimental groups were compared to the negative control group. *P < 0.05, ^#^*P* < 0.01, ^+^*P* < 0.001.

To explore the signaling downstream of ACh, we analyzed stomach adenocarcinoma data from the TCGA dataset and discovered that the level of AMPK phosphorylated at Thr 172 is positively correlated with expression of CHRM3 mRNA, which encodes M3R (Figure 3A). Moreover, it was verified in an earlier study in our laboratory that MACC1 can be up-regulated by p-AMPK [19]. We therefore speculated that MACC1 may mediate the stimulatory effects of ACh on GC cell invasion/migration and EMT. To test that idea, we examined the expression levels of MACC1 mRNA and protein in MKN45 and MGC803 cells after ACh stimulation. As anticipated, ACh time-dependently up-regulated MACC1 expression (Figure 3B and 3C), and this effect was suppressed by darifenacin (Figure 3D and 3E). However, ACh-induced nuclear translocation of MACC1 was not significant (Supplementary Figure 2). Thereafter, MKN45 and MGC803 cells were transfected with MACC1 siRNA (siMACC1) or control siRNA (siCtrl) and then treated with or without ACh for 48 h. The knockdown efficiency of siMACC1 was determined using qRT-PCR and western blotting (Figure 4A and 4B). As shown in Figure 4C and 4D, the ACh-induced increase in GC cell invasion/migration was restrained after MACC1 knockdown. The same trend was observed for the mRNA and protein expression of EMT makers (Figure 4E and 4F). These findings suggest that MACC1 actively mediates ACh-induced GC cell invasion/migration and EMT.

### MACC1 is up-regulated by ACh through p-AMPK

**Figure 5:**
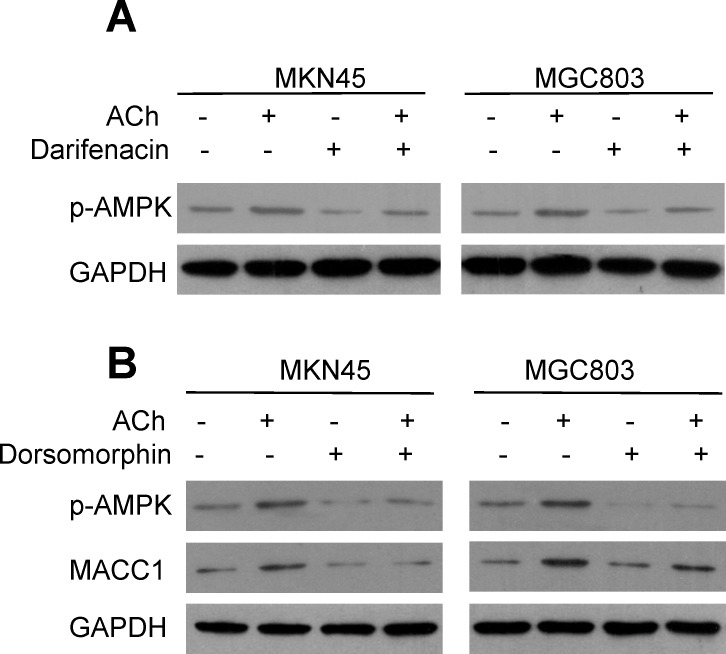
MACC1 is up-regulated by ACh through p-AMPK **A.** ACh stimulated AMPK phosphorylation via M3R. **B.** Inhibition of AMPK activity by dorsomorphin (8 μM) suppressed the induction of MACC1 expression by ACh.

To further confirm that p-AMPK is involved in the positive regulation of ACh/M3R on MACC1 expression, we treated MKN45 and MGC803 cells with ACh and then used western blotting to assess the p-AMPK levels. The results showed that p-AMPK levels increased after ACh stimulation, and pretreatment of darifenacin attenuated the ACh-induced increase of p-AMPK (Figure 5A). This indicates that p-AMPK is a downstream signal for M3R in GC cells. When MKN45 and MGC803 cells were treated with the p-AMPK inhibitor dorsomorphin, ACh-stimulated up-regulation of MACC1 was greatly inhibited (Figure 5B). It thus appears ACh acts via M3R and p-AMPK to up-regulate MACC1.

## DISCUSSION

It has long been recognized that perineural invasion (PNI), defined as the process in which tumor cells invade the perineural space of nerves, serves an important role in tumor metastasis [20, 21]. Until recently, however, little attention was paid to the effect of peripheral nerve infiltration on tumor development and progression. One important study revealed that the sympathetic and parasympathetic nervous systems are indispensable for prostate cancer progression in mice and that they act in concert to simulate prostate cancer growth and metastasis [1]. More recently, Zhao et al showed that surgical or pharmacological denervation of the stomach suppressed gastric tumor development and progression [2]. In the same study, they showed that vagotomy inhibited gastric Wnt signaling and suppressed stem cell expansion mediated via M3Rs, which were most likely stimulated by ACh released by the vagal nerve.

Several studies have reported on the roles of ACh and M3Rs in cancer. In non-small cell lung cancer cells, for example, M3Rs activated by ACh promote cell proliferation and invasion via the EGFR/PI3K/AKT pathway [10], while in colon cancer M3R activation exerts a growth-promoting effect by mediating activation of the p21Ras-ERK pathway through tyrosine-phosphorylated EGFR [8]. In addition, the CaM/CaMKK/p-Akt axis reportedly plays an important role in M3R-mediated autocrine promotion of prostate cancer growth and castration resistance [22].

Research into the role played by M3Rs in GC date back to a study done in the late 1990s [23]. In that study, carbachol activation of M3Rs stimulated tyrosine phosphorylation of mitogen-activated protein kinase (MAPK), but evoked no obvious DNA synthesis or cell proliferation. By contrast, Wang et al. very recently demonstrated that ACh secreted by GC cells acts via M3Rs to stimulate GC cell proliferation, and that M3Rs are highly expressed in GC tissues, which is related to tumor stage and lymph node metastasis [24]. Our findings in the present study verify that ACh promotes GC cell invasion/migration as well as expression of EMT markers, and that those effects can be inhibited using a M3R antagonist (darifenacin). It is also noteworthy that even in the absence of exogenous ACh, M3R blockade suppressed GC cell invasion/migration and EMT. We therefore speculate that MKN45 and MGC803 GC cells may secrete endogenous ACh, which then activates M3Rs in an autocrine fashion. This finding is consistent with the results of Wang et al. [24].

Ours is the first observation that the oncogene MACC1 functions as a downstream signal for ACh, which acted via M3Rs to promote MACC1 expression. Moreover, MACC1 knockdown significantly attenuated the effects of ACh on GC cell invasion/migration and expression of EMT markers. While these findings are consistent with MACC1 serving as an intracellular signal downstream of extracellular ACh, they do not preclude the existence of other oncogenes or signaling pathways mediating ACh-induced GC cell migration/invasion. It remains to be determined whether silencing MACC1 *in vivo* suppresses the effect of ACh or vagal nerve activity on GC.

In an earlier study in our laboratory, Wang et al. showed for the first time that MACC1 is highly expressed in human GC and that it promotes GC cell proliferation, invasion and EMT [16]. Lin et al. subsequently showed that by enhancing the Warburg effect, MACC1 plays a significant role in supporting GC cell survival and proliferation during glucose deprivation-induced metabolic stress [19]. In addition, MACC1 reportedly promotes lymphangiogenesis in GC by up-regulating vascular endothelial growth factor-C/D [25] and vasculogenic mimicry by up-regulating TWIST1/2 [26]. We confirmed here that ACh, acting via M3Rs, increases expression of MACC1 through phosphorylation (activation) of AMPK. Taken together with the results of the previous studies in our laboratory, our present findings suggest that extracellular ACh induces up-regulation of intracellular MACC1, which in turn promotes lymphangiogenesis and vasculogenic mimicry in the tumor microenvironment, thereby promoting GC growth and metastasis. The involvement of AMPK in that process implies that neurotransmitter ACh has the potential to regulate cellular metabolism via the energy sensor AMPK, though this needs to be investigated further.

In summary, our study verified that ACh promotes invasion/migration of GC cells, and that the M3R/AMPK/MACC1 signaling pathway plays a pivotal role. These findings increase our understanding of how MACC1 is up-regulated and sheds light on a potential new therapeutic target for the treatment of GC.

## MATERIALS AND METHODS

### Materials

Anti-MACC1, Anti-E-cadherin and anti-vimentin antibodies were obtained from Abcam (Cambridge, UK). Anti-MMP2, anti-p-AMPK, anti-MMP9 and anti-fibronectin antibodies were purchased from Novus Biologicals (Colorado, USA), Signalway Antibody (Maryland, USA) and Proteintech (Chicago, USA), respectively. Goat anti-rabbit IgG and rabbit anti-mouse IgG for western blotting were from SouthernBiotech (AL, USA). Alexa Fluor 448-labeled antibodies for immunofluorescence assays were from Beyotime (ShangHai, China). ACh was purchased from BioBasic Inc. (Toronto, Canada). Darifenacin and dorsomorphin were provided by MedChemExpress (NJ, USA). Lipofectamine 2000 reagent was from Invitrogen (CA, USA), while a Trizol kit was from Takara (Tokyo, Japan). Triton, bovine serum albumin (BSA) and DAPI were from DINGGUO CHANGSHENG (Beijing, China), Biosharp (AnHui, China) and Beyotime (ShangHai, China), respectively.

### Cell culture

The MGC-803 and MNK-45 human GC cell lines were obtained from Foleibao Biotechnology Development Co. (Shanghai, China). The cells were cultured in RPMI-1640 medium with 10% fetal bovine serum (Thermo Scientific HyClone, USA) at 37°C under 5% CO_2_.

### Migration and invasion assays

Cell migration was evaluated using transwell migration assays with non-coated inserts, while cell invasion was assessed in transwell invasion assays with Matrigel-coated inserts. For each transwell migration assay, 5×10^4^ cells suspended in 200 μL of serum-free RPMI-1640 were seeded into the upper chamber (Corning Incorporated, NYC, USA) of a non-coated transwell insert. For each transwell invasion assay, the cells were seeded into the upper chamber an insert coated with 100 μL of 10% Matrigel (Corning Incorporated, NYC, USA). In both assays, the lower compartment was filled with 600 μL RPMI-1640 containing 10% fetal bovine serum as a chemoattractant. After incubation for 24 h 37°C in 5% CO_2_, the incubation medium and the cells on the upper surface of membrane, which did not migrate or invade, were removed with cotton swabs and phosphate buffered saline (PBS). Cells on the underside of the membrane were fixed in 4% paraformaldehyde, stained with 0.1% crystal violet, photographed under an inverted microscope at 200x magnification and counted offline.

### Quantitative real-time PCR

Total RNA was extracted from cultured cells using a Trizol kit according to the manufacturer's instructions and then reverse transcribed using Takara RT reagent. The primer sequences used for real-time PCR are listed in Table 1. Expression of candidate genes was normalized to that of GAPDH. Quantitative real-time PCR were performed using a LightCycler 480 system Version 1.5 (Roche, Penzberg, Germany). Samples were run and calculated in triplicate.

**Table 1:** Real-time PCR primers

Gene	Sequence (5′-3′)
E-cadherin-F	ATGAGTGTCCCCCGGTATCT
E-cadherin-R	CAAACACGAGCAGAGAATCA
Vimentin-F	CGCCAGATGCGTGAAATGG
Vimentin-R	ACCAGAGGGAGTGAATCCAGA
Fibronectin-F	ATGATGAGGTGCACGTGTGT
Fibronectin-R	CCCTGACCGAAGCATGTACA
MMP2-F	CTGGAGATACAATGAGGTGAAG
MMP2-R	TCTGAGGGTTGGTGGGATTG
MMP9-F	TACCACCTCGAACTTTGACA
MMP9-R	AGGGCGAGGACCATAGAG
MACC1-F	ATCCGCCACACATGCTTAA
MACC1-R	CTTCAGCCCCAATTTTCATC
GAPDH-F	ACCCAGAAGACTGTGGATGG
GAPDH-R	TCTAGACGGCAGGTCAGGTC

### Western blot analysis

Cells were washed with cold PBS and homogenized in lysis buffer containing protease inhibitors (keyGEN, Nanjing, China) on ice. After centrifugation, the protein-containing supernatant was collected. Total protein and 5x SDS loading buffer were mixed and boiled at 100°C for 5 min. Samples were separated by electrophoresis on 10% SDS-polyacrylamide gel and transferred onto polyvinylidene fluoride membranes, after which the membranes were blocked for 1 h at room temperature with 5% skim milk supplemented with 0.1% Tween 20 (TBST). Each membrane was then first incubated overnight with a primary antibody at 4°C and then with a secondary antibody for 120 min at room temperature. Immunoreactive bands were visualized using a chemiluminescence (ECL) detection system.

### Small interfering RNA transfection

MGC-803 and MNK-45 GC cells were seeded into six-well plates. Once the cells reached 90% confluence, they were transfected with small interfering RNA (siRNA) targeting MACC1 or with Control siRNA using Lipofectamine 2000 reagent as instructed by the manufacturer. The sequences of MACC1 siRNA were 5′-GCCACAAGAUUUAAGUAUUdTdT-3′ and 3′-dTdTCGGUGUUCUAAAUUCAUAA-5′. The knockdown efficiency was verified using quantitative real-time PCR and western blotting.

### Immunofluorescence assay

Cells on small culture dishes were fixed with 4% paraformaldehyde for 15 min at room temperature and permeabilized with 0.5% Triton, after which they were washed three times with PBS and blocked with 5% BSA for 30 min. The cells were then incubated overnighted at 4°C with primary anti-MACC1 antibody (1:100), rinsed and incubated for 1 h at room temperature with Alexa Fluor 448-labeled second antibodies. The cells were then washed three times with PBS, and the nuclei were staining for 5 min with 5 μg/ml DAPI. Fluorescence images were obtained using a confocal laser scanning microscope (Olympus, Japan).

### Bioinformatics analysis

To analyze the correlation between the levels of CHRM3 mRNA and AMPK phosphorylation in gastric adenocarcinoma, we retrieved and analyzed data from the TGCA dataset using a tool at http://www.cbioportal.org/. Specifically, we selected the “Stomach Adenocarcinoma (TGCA, Provisional)” database for further query. “mRNA Expression z-Store (RNA Seq RPKM)” and “protein/phosphoprotein level (RPPA)” were selected in the “Select Genomic Profiles” section. In the “Enter Gene set” dialog box, we input our command as “CHRM3: EXP < 0”. On the following page, the corresponding figure, which showed the change in the AMPK phosphorylation level when CHRM3 mRNA expression was altered, could be depicted after clicking “AMPK (T172)” tab listed below the “protein change” module.

### Statistical analysis

All statistical analyses were performed using SPSS software version 20.0 (SPSS Inc., Chicago, IL, USA). Experiments were repeated at least three times. Data are presented as the mean ± SEM. Differences between groups were analyzed using one-way ANOVA. Two-sided p values less than 0.05 were considered significant.

## SUPPLEMENTARY MATERIAL FIGURES


