# Supplementary material for: MACC1 mediates acetylcholine-induced invasion and migration by human gastric cancer cells
Source: Oncotarget. 2016 Feb 23;7(14):18085–94. doi: 10.18632/oncotarget.7634 (PMC4951273; doi:10.18632/oncotarget.7634)
Supplement: Supplementary file 1 [file oncotarget-07-18085-s001.pdf]

MACC1 mediates acetylcholine-induced invasion and migration by human gastric cancer cells

Supplementary Material

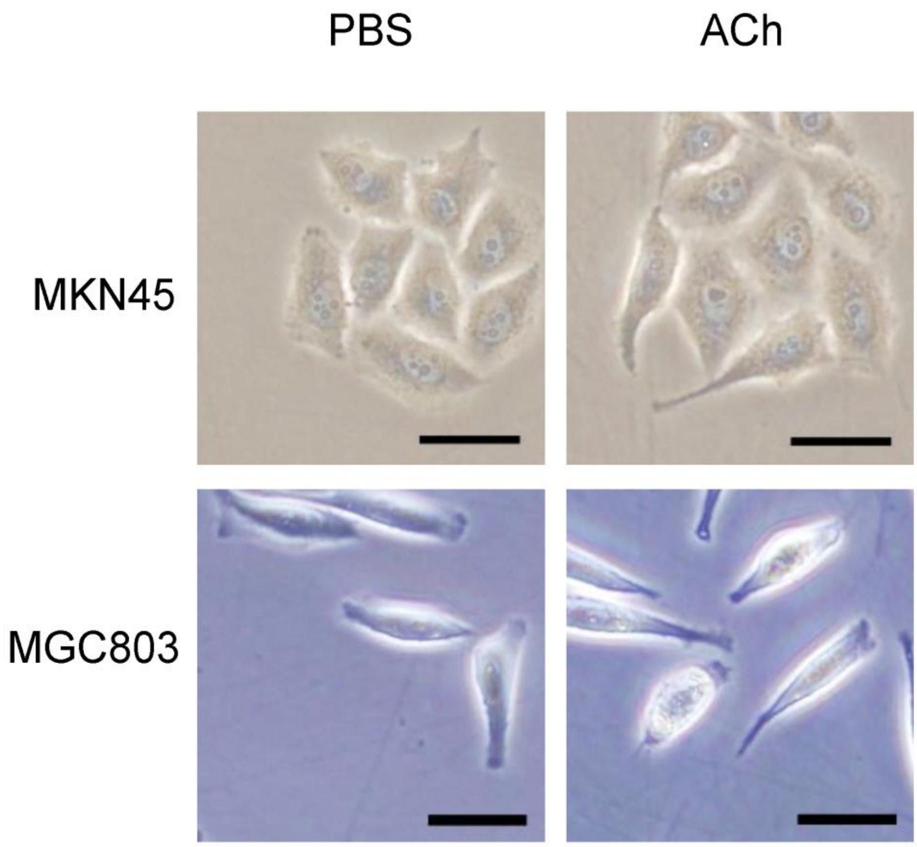

Supplementary Figure 1. ACh does not induce significant morphological changes in gastric cancer cells. MKN45 and MGC803 cells were incubated for 48h with ACh (10 μM). PBS was used as a negative control. Cell morphology was photographed under an ordinary optical microscope. Scale bar = 50 μm.

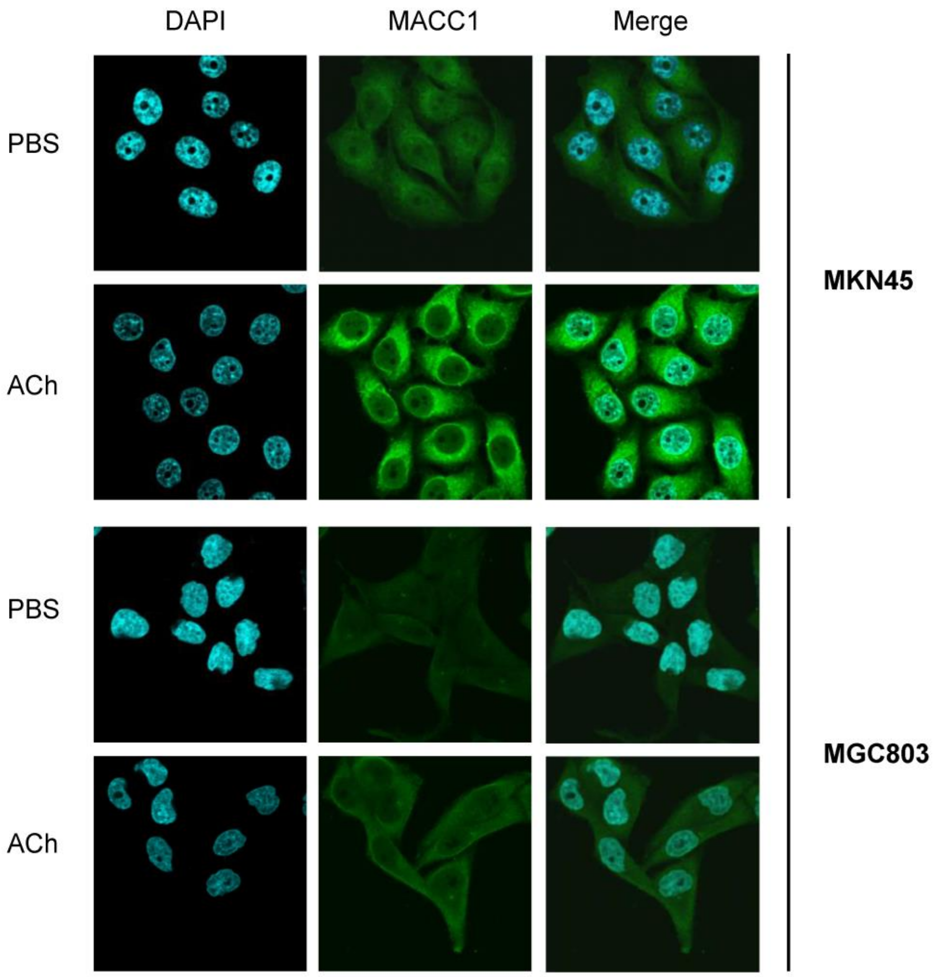

Supplementary Figure 2. ACh promotes no significant translocation of MACC1. MKN45 and MGC803 cells were incubated for 48h with ACh (10 μM). PBS was used as a negative control. Immunofluorescent staining showed that MACC1 (green) levels were higher after ACh stimulation than in the negative control, but there was no significant nuclear translocation of MACC1. Magnification: 120x.
